# Immobilization of ZnO-TiO_2_ Nanocomposite into Polyimidazolium Amphiphilic Chitosan Film, Targeting Improving Its Antimicrobial and Antibiofilm Applications

**DOI:** 10.3390/antibiotics12071110

**Published:** 2023-06-27

**Authors:** Wesam Abd El-Fattah, Mohammad Y. Alfaifi, Jafar Alkabli, Heba A. Ramadan, Ali A. Shati, Serag Eldin I. Elbehairi, Reda F. M. Elshaarawy, Islam Kamal, Moustafa M. Saleh

**Affiliations:** 1 Chemistry Department, College of Science, IMSIU (Imam Mohammad Ibn Saud Islamic University), P.O. Box 5701, Riyadh 11432, Saudi Arabia; wabdulfatah@imamu.edu.sa; 2 Department of Chemistry, Faculty of Science, Port Said University, Port Said 42521, Egypt; 3 Biology Department, Faculty of Science, King Khalid University, Abha 61413, Saudi Arabia; aaalshati@kku.edu.sa (A.A.S.); serag@kku.edu.sa (S.E.I.E.); 4 Department of Chemistry, College of Sciences and Arts—Alkamil, University of Jeddah, Jeddah 23218, Saudi Arabia; jabdalsamad@uj.edu.sa; 5 Department of Microbiology and Immunology, Faculty of Pharmacy, Delta University for Science and Technology, Mansoura 11152, Egypt; hebaaa.aadel@gmail.com; 6 Department of Chemistry, Faculty of Science, Suez University, Suez 43533, Egypt; 7 Institute for Inorganic Chemistry and Structural Chemistry, Düsseldorf University, 40225 Düsseldorf, Germany; 8 Department of Pharmaceutics, Faculty of Pharmacy, Port Said University, Port Said 42526, Egypt; islamkamal@pharm.psu.edu.eg; 9 Microbiology and Immunology Department, Faculty of Pharmacy, Port Said University, Port Said 42526, Egypt; mostafa.mohamed@pharm.psu.edu.eg

**Keywords:** polyimidazolium amphiphilic chitosan Schiff base film, hybrid ZnO-TiO_2_ nanocomposite, antimicrobial, antibiofilm, cytotoxicity

## Abstract

This study presents a green protocol for the fabrication of a multifunctional smart nanobiocomposite (NBC) (ZnO-PIACSB-TiO_2_) for secure antimicrobial and antibiofilm applications. First, shrimp shells were upgraded to a polyimidazolium amphiphilic chitosan Schiff base (PIACSB) through a series of physicochemical processes. After that, the PIACSB was used as an encapsulating and coating agent to manufacture a hybrid NBC in situ by co-encapsulating ZnONPs and TiO_2_NPs. The physicochemical and visual characteristics of the new NBC were investigated by spectral, microscopic, electrical, and thermal methods. The antimicrobial indices revealed that the newly synthesized, PIACSB-coated TiO_2_–ZnO nanocomposite is an exciting antibiotic due to its amazing antimicrobial activity (MIC/MBC→0.34/0.68 μg/mL, 0.20/0.40 μg/mL, and 0.15/0.30 μg/mL working against *S. aureus*, *E. coli*, and *P. aeruginosa*, respectively) and antifungal capabilities. Additionally, ZnO-PIACSB-TiO2 is a potential fighter of bacterial biofilms, with the results being superior to those of the positive control (Cipro), which worked against *S. aureus* (only 8.7% ± 1.9 biofilm growth), *E. coli* (only 1.4% ± 1.1 biofilm growth), and *P. aeruginosa* (only 0.85% ± 1.3 biofilm growth). Meanwhile, the NBC exhibits excellent biocompatibility, as evidenced by its IC_50_ values against both L929 and HSF (135 and 143 µg/mL), which are significantly higher than those of the MIC doses (0.24–24.85 µg/mL) that work against all tested microbes, as well as the uncoated nanocomposite (IC_50_ = 19.36 ± 2.04 and 23.48 ± 1.56 µg/mL). These findings imply that the new PIACSB-coated nanocomposite film may offer promising multifunctional food packaging additives to address the customer demand for safe, eco-friendly food products with outstanding antimicrobial and antibiofilm capabilities.

## 1. Introduction

Antibiotic resistance (ABR) and antibiotic-adverse drug reactions (ADRs) are significant problems in public health, leading to prolonged and costly treatments, increased morbidity and mortality rates, and reduced quality of life among patients. ABR occurs when infected cells develop immunity to numerous medicines. This phenomenon has become a significant public health concern, as it leads to the failure of treatments and increases the morbidity and mortality rates of infectious diseases. There are various causes of ABR, including the overuse of drugs, the overexpression of efflux pumps, alterations in drug targets or pathways, changes in drug metabolism, and genetic mutations [1,2]. On the other hand, ADRs are a major concern in clinical practice, especially in elderly patients with multiple comorbidities and polypharmacy circumstances. ADRs can be classified into two groups, Type-A and Type-B. Type-A reactions are predictable, dose-dependent, and related to the pharmacological properties of the drug, whereas Type-B reactions are unpredictable, dose-independent, and are not related to the pharmacological properties of the drug. Type-B reactions are often immune-mediated, idiosyncratic, or genetic in nature [3,4,5]. To tackle these challenges, innovative approaches are being developed and implemented. One such approach is the use of precision medicine. Precision medicine involves tailoring the treatment to an individual’s genetic makeup, lifestyle, and environment. This approach can help healthcare providers identify which antibiotics will work best for a particular patient, reducing the risk of antibiotic resistance and adverse drug reactions [6]. Another promising approach is the development of new antibiotics and alternative treatments. Scientists are exploring new compounds and technologies to create antibiotics that are effective against resistant bacteria. For example, researchers are investigating the use of bacteriophages, which are viruses that can infect and kill bacteria. They are also exploring the use of probiotics, which are live microorganisms that can restore the balance of bacteria in the gut and reduce the risk of infections [7,8].

Bacterial biofilm formation is a complex process that involves the adherence of bacterial cells to a surface and the development of a three-dimensional matrix of extracellular polymeric substances. This matrix serves as a protective barrier against environmental stresses, such as antibiotics and disinfectants, thereby promoting ABR [9]. In addition, biofilms can also have negative impacts on human health. Targeting and inhibiting biofilm formation is important for preventing these issues. The formation of biofilms is regulated by a quorum-sensing (QS) mechanism involving signal molecules, which is essential for the formation of biofilms in many bacterial species, including *P. aeruginosa*, *E. coli*, and *S. aureus* [10]. One promising approach to preventing biofilm growth is the inhibition of signal molecules, such as QS molecules. Other approaches have been developed to eradicate bacterial biofilms, including the use of monoclonal antibodies that can target specific surface proteins on bacteria, preventing them from adhering to surfaces and forming biofilms; the use of enzymes that degrade the extracellular matrix; the use of bacteriophages that specifically target the bacterial host; and the use of nanoparticles that can penetrate the biofilm matrix and disrupt the biofilm structure [11,12].

Despite the availability of multiple strategies intended to overcome ABR and ADR issues, such as combination therapies, the development of new antibiotics, and targeted therapy [13,14], the optimal approach to addressing ABR and ADRs has not yet been attained. In this context, pharmaceutical researchers have been striving to develop effective nanostructured delivery systems and microstructured drug delivery systems (NDDSs and MDDSs) to enhance the therapeutic outcomes of antibiotics, as well as to tackle the current challenges of ABRs, ADRs, and the low aqueous solubility of the drug [15]. In recent years, metals and metal oxide nanoparticles (MNPs/MONPs) have gained increasing attention in the field of biomedicine regarding their ability to reduce ABRs and ADRs due to their unique physicochemical and biological properties [16,17]. One promising approach is the use of MONPs to enhance the effectiveness of existing antibiotics. MONPs have been shown to possess antimicrobial properties and can enhance the penetration of antibiotics into bacterial cells by disrupting the bacterial membrane. In addition, MONPs can also inhibit bacterial efflux pumps, which are a major mechanism of antibiotic resistance. Therefore, the use of MONPs in combination with antibiotics resulted in a significant reduction in the minimum inhibitory concentration (MIC) of several antibiotics acting against resistant strains of bacteria [18,19]. Particularly, TiO_2_NPs and ZnONPs have been singled out as two of the most promising MONPs for use as antimicrobials. TiO_2_NPs and ZnONPs are promising antimicrobials due to their physicochemical properties that make them effective against a wide range of microorganisms. TiO_2_NP is an excellent photocatalyst that can effectively generate reactive oxygen species (ROS) when exposed to light, which can damage the cell membrane of microorganisms and lead to their death. In addition, the ultra-small size of TiO_2_NPs and ZnONPs enhances their ability to interact with bacteria and viruses. On the other hand, ZnONPs are effective antimicrobials due to their high surface area and their ability to release zinc ions that can disrupt the cell membrane of microorganisms [20,21,22]. However, the potential toxicity and environmental risks of these nanoparticles are major concerns that need to be addressed for safe biomedical applications [23]. Several strategies have been proposed to mitigate the potential toxicity of TiO_2_NPs and ZnONPs, such as surface modifications, size and shape control, the use of natural antioxidants, chelation therapy, and encapsulation by biocompatible materials [20,24].

The encapsulation of these MONPs with biocompatible and biodegradable biopolymers is considered a promising strategy to mitigate their potential toxicities. The use of biopolymers in encapsulation has been shown to improve the stability, bioavailability, and therapeutic efficacy of MONPs. These biopolymers provide a protective barrier that limits the interaction of the nanoparticles with living organisms, reducing their adverse effects. Furthermore, encapsulation can improve the pharmacokinetics of NPs by increasing their circulation time in the bloodstream, thus enhancing their therapeutic efficacy [25,26,27]. Amongst encapsulating materials, chitosan-based materials are the best choice because of their superior properties and potential, including their antimicrobial and antioxidant efficacy, biodegradability, biocompatibility, nontoxicity, chelating abilities, and film-forming capabilities [25,28]. Polyimidazolium chitosan Schiff bases (PICSBs) are novel and biocompatible materials that have recently gained attention for their potential applications in various fields, such as biomedicine, water treatment, and energy storage. PICSBs exhibit excellent thermal stability and remarkable mechanical properties, making them ideal coating stabilizing agents for metallic nanoparticles. Furthermore, PICSBs have been shown to exhibit antibacterial and antifungal activities, making them potential candidates for use in biomedical and food-packaging applications. Overall, the unique properties of PICSBs make them promising materials for a wide range of applications; further research in this area is needed to fully explore their potential [29].

These remarkable findings served as our inspiration and as a new step in our ongoing exploration and development of innovative pharmacological substances [30,31,32]. The objective of the current study is to create a novel polyimidazolium-supported amphiphilic chitosan Schiff base (PIACSB) that can tightly adhere and co-encapsulate a ZnONP and TiO_2_NP cocktail in situ to create ternary nanobiocomposite (NBC) (ZnO-PIACSB-TiO_2_) for safe antimicrobial and antibiofilm applications.

## 2. Results and Discussion

### 2.1. Chemistry of Preparation Processes

**Figure 1:**
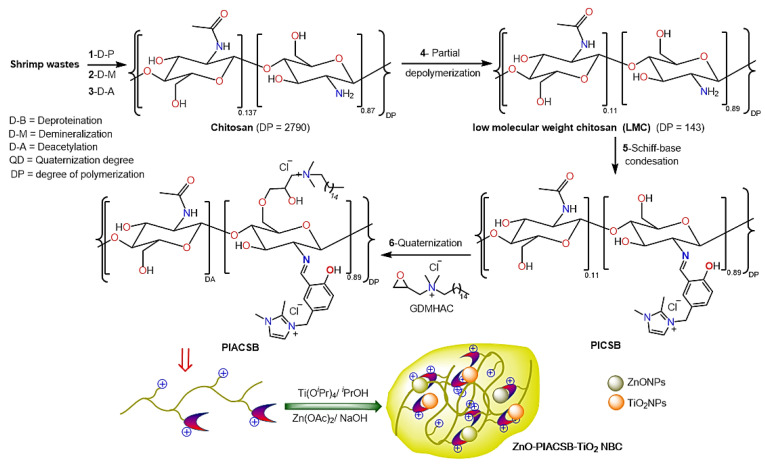
Step-by-step synthesis of PIACSB and ZnO-PIACSB-TiO_2_ NBC.

As shown in Figure 1, a series of consecutive physical processes and chemical reactions were used to upgrade the shrimp shells to the PIACSB. In the pathway, chitosan was extracted from the shrimp shells through several consecutive steps, including deproteinization (to remove proteinaceous materials from shrimp shells) by treatment with NaOH solution, demineralization (to remove insoluble carbonates) by treatment with HCl solution, and deacetylation by treatment with NaOH solution, to convert the obtained chitin to chitosan. Afterward, the partial degradation of chitosan by treatment with H_2_O_2_ yielded the corresponding low molecular weight chitosan (LMC). In another route, the salicylaldehyde-functionalized imidazolium ionic liquid (SIIL) was prepared by the chloromethylation of salicylaldehyde, followed by the quaternization of 1,2-dimethylimidazole, to give SIIL. A Schiff base condensation reaction was performed between LMC and SIIL to produce a polyimidazolium-tagged chitosan Schiff base (PICSB) that was subjected to an O-quaternization process with GDMHAC to form the intended PIACSB. Eventually, the PIACSB was applied as an encapsulating agent during the in situ preparation of ternary the NBC (ZnO-PIACSB-TiO_2_) by the co-precipitation of ZnONPs while preparing TiO_2_NPs; this process used a sol-gel method from their respective precursors, mediated by ultrasonic irradiation in a PIACSB dispersion. An aqueous NaOH solution was used as a gelling and precipitating agent in this technique.

### 2.2. Physicochemical Characterization

#### 2.2.1. Physical Characterization

The average molecular weight (*M_av_*) of LMC, PACSB, and PIACSB solutions in aqueous NaCl (0.1 M) was determined by measuring their intrinsic viscosity “*η*” and applying the Mark–Houwink–Sakurada formula (Equation (1)) [33]:

[*η*] = *K* (*M_av_*)^*α*^ (1)

The *M_av_* for the LMC, PACSB, and PIACSB samples were found to be 24.48, 35.42, and 49.05 KDa, respectively, which are equivalent to degrees of polymerization (DP) of ~143, ~140, and ~138, respectively.

The zeta “ζ” potential of a nanocomposite is an important factor that affects its physical stability and performance in aqueous media. The ζ-potential of this nanocomposite is known to have a significant impact on its properties and performance, including its stability, dispersibility, and antibacterial activity. Table S1 (Supplementary Materials) shows that the NBC and its encapsulating agent (PIACSB) both have extremely high positive ζ-potential values (PIACSB, +56.81 mV; NBC +47.22 mV), well over the characterized stability limit for nanoformulations (30 mV) [34], which is in line with their cationic nature.

**Figure 2:**
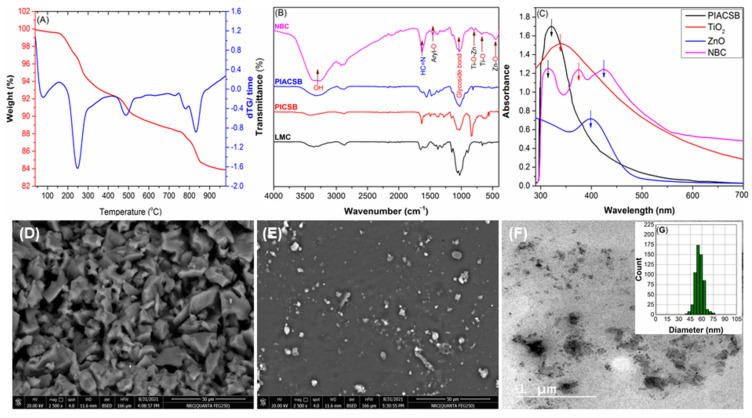
(**A**) Thermogravimetric analysis (TGA) and differential thermal analysis (DTA) curves of the ZnO-PIACSB-TiO_2_ NBC. (**B**) FTIR spectra of newly produced materials displaying the key, recognizable absorption bands. (**C**) UV-Vis spectra of the obtained materials displaying the key, recognizable electronic transition peaks. (**F**) SEM microimages of (**D**) PIACSB and (**E**) ZnO-PIACSB-TiO_2_ NBC. (**F**) TEM nanoimage of the ZnO-PIACSB-TiO_2_ NBC. (**G**) PSD histograms of the ZnO-PIACSB-TiO_2_ NBC. Arrows in (**C**) refer to the characteristic functional groups of each material.

On the other hand, thermogravimetric analysis (TGA) and differential thermal analysis (DTA) are essential tools in nanocomposite characterization that have been used to determine its thermal stability, composition, and degradation kinetics. According to the TGA and DTA thermograms (Figure 2A), the ZnO-PIACSB-TiO_2_ NBC is thermally stable up to ~250 °C; however, ultimately it undergoes degradation through three thermal decomposition stages. Initially, the NBC lost the physically adsorbed water within the temperature range of 43–127 °C, with an endothermic maximum of 78 °C and a 2.77% weight loss. The first (241–453 °C, endothermic maxima at 249 °C) and second (476–770 °C, endothermic maxima at 486 °C) decomposition stages with major mass losses could be attributed to the degradation of the PIACSB chain, which occurred by the initial loss of the segments grafted on the LMC surface and then the decomposition of the chitosan chain. The last endothermic event, which occurred between 775 °C and 966 °C at a peak of 832 °C, could be attributed to the complete de-encapsulation of the ZnO-PIACSB-TiO_2_ NBC and the formation of a ZnO-TiO_2_ nanocomposite [32].

#### 2.2.2. Structural Characterization

Initially, the titrimetric and elemental analyses (EA) results were used. As described in our earlier work [29,33], we first used the results from titrimetric and elemental analyses (EA) to determine the degrees of deacetylation (DD%) in LMC, grafting (GD%) in PICSB, and quaternization (QD%) in PIACSB. The results from these computations are shown in Table S1 (Supplementary Materials). It is noteworthy that there are remarkable variations between the values of nitrogen-to-carbon ratios in the LMC (0.195), PICSB (0.183), and PIACSB (0.117) that are indicative that the grafting and quaternization processes were successful. In addition, the EA results for PICSB and PIACSB (Table S1, Supplementary Materials) further suggest partial grafting and quaternization processes because they do not agree with the full Schiff base condensation of the NH_2_ group of LMC with SIIL or the quaternization of the OH group of PICSB with the GDMHAC fragment.

On the other hand, energy-dispersive X-ray spectroscopy (EDS) was used to qualitatively and quantitatively estimate the elemental composition of PICSB and its ternary nanobiocomposite (ZnO-PIACSB-TiO_2_) at a microscopic level. The results of the EDS analysis showed that the PIACSB (Figure S1, Supplementary Materials) contains elemental carbon, oxygen, nitrogen, and chlorine. The presence of C, N, and O is due to both the chitosan chain and salicylidene imidazolium brushes, while the Cl is due to the imidazolium ionic liquid. On the other hand, the EDS analysis of the NBC (Figure S2, Supplementary Materials) displayed new titanium and zinc peaks in addition to the native C, N, O, and Cl peaks; however, this resulted in different percentages, as indicative of the homogeneous distribution of TiO_2_NPs and ZnONPs into the PIACSB network.

Comparing the FTIR spectrum of PIACSB (Figure 2A) with those of its native components (LMC, SIIL, and PICSB) (Figures S3–S5, Supplementary Materials) provided another element of the proof of its successful production. The NH_2_ bending absorption peak (1588 cm^−1^) is still present in the PICSB spectrum, albeit with a much weaker intensity than in the LMC spectrum; two additional peaks appear at 1618 and 1271 cm^−1^ that may be attributed to the vibration of the azomethine and aryl-O groups, respectively. Additionally, the characteristic carbonyl peak of the SIIL (1669 cm^−1^) nearly vanished in the PICSB spectrum. According to these results, the PICSB is formed by the partial Schiff base condensation reaction of LMC with SIIL [35]. Meanwhile, the presence of vibration bands typical of the PICSB (3348 cm^−1^ for O-H/N-H, 1618 cm^−1^ for azomethine, 1271 cm^−1^ for aryl-O, and 836 cm^−1^ for the glycoside group) and the GDMHAC segment (2961 and 2883 cm^−1^ for the methyl groups; 1510 and 667 cm^−1^ for R_4_N^+^) in the spectrum of PIACSB (Figure 2B), albeit with remarkable changes in their intensities and/or sites due to the quaternization reaction, is indicative of the successful formation of the PIACSB [33]. As for the NBC, the immobilization of ZnO and TiO_2_ into the matrix of the PIACSB, as well as the mode of interactions between the PIACSB polymeric chain and inorganic nanoparticles (ZnO and TiO_2_), can be made evident by the significant effects of these nanoparticles on the characteristic FTIR peaks of the PIACSB. As can be seen in Figure 2B, the intermolecular interactions between the PIACSB and ZnO-TiO_2_ nanoparticles are validated by the alteration of the main characteristics (intensity and site) of the key peaks for the functional groups of the PIACSB (OH, NH, azomethine, and aryl-O). Among other things, the O-H peak was moved from 3346 cm^−1^ in the PIACSB spectrum to 3321 cm^−1^ in the NBC spectrum. Additionally, the nanocomposite spectrum showed a negative shift of about −9 cm^−1^ in the azomethine peak. Moreover, the site of the aryl-O peak has been found to have shifted negatively (∆ν = −12 cm^−1^). These results demonstrate that interactions with ZnO and TiO_2_ share hydroxyl, azomite, and phenolic groups [36]. Additional proof that metallic nanoparticles have adhered to the PIACSB surface was provided by the appearance of new FTIR peaks in the spectra of the nanocomposite at 853, 719, and 506 cm^−1^, which are characteristic of the Zn-O-Ti, Ti-O, and Zn-O, respectively [36].

To further validate the immobilization of the ZnO and TiO_2_ nanoparticles into the PIACSB matrix, UV-Vis spectroscopy was utilized, which is a powerful tool to provide valuable insight into the NBC’s optical properties. To proceed with this, we obtained ZnO and TiO_2_ nanoparticles from our previous work [32] and compared the UV spectrum of the NBC (ZnO-PIACSB-TiO_2_) to those of native components (PIACSB and nascent nanoparticles) (see Figure 2C). Two absorption bands, one faint at 298 nm and one strong at 319 nm, were seen in the PIACSB spectrum. These bands might be attributed to the π→π* and n→π* transitions of the benzene ring and the carbonyl/azomethine groups of the chitosan and the salicylidene fragment, respectively [35,37]. When comparing the UV spectrum of the NBC with those of its native ingredients, it can be seen that the NBC spectrum combines the characteristic absorption peaks of PIACSB, TiO_2_NPs, and ZnONPs; however, this takes place with blue or red shifts as a result of their mutual interactions. Noticeably, the NBC spectrum exhibited two absorption bands at 295 and 315 nm, which were attributed to the electronic transitions in the PIACSB (blue-shifted). In addition, the peaks observed at 367 and 439 nm could be attributed to the bandgap absorption of the TiO_2_–ZnO nanocomposite. The red shifts in the bandgap absorption of the nanocomposite indicated the formation of a heterojunction between TiO_2_ and ZnO, leading to an increase in the separation of photogenerated electron-hole pairs [38]. These results prove that a nanocomposite of PIACSB and metallic oxide was successfully formed by coating a TiO_2_-ZnO nanocomposite with PIACSB film.

Multiple configurations of proton peaks can be seen in the ^1^H NMR spectrum of PIACSB (Figure S6, Supplementary Materials). The first set of proton configurations (singlet and multiplet peaks) can be seen in the low-field region (10.19–7.34 ppm) of the PIACSB spectrum, assignable to the nuclear resonances of intramolecular H-bonded phenolic OH, azomethine, salicylidene, and imidazolium protons. The other two sets of proton configurations derived from the resonances of the protons belong to the chitosan chain; the GDMHAC segment can be seen in the high-field region (5.13–1.12 ppm) as very crowded proton signals with various splitting patterns and chemical shifts due to the huge number of protons in various chemical environments. Further evidence for chemical modification of the LMC skeleton with SIIL and GDMHAC, while maintaining the structural identity of the chitosan chain, is provided by the ^13^C NMR spectra of the PIACSB (Figure S7, Supplementary Materials). In this context, the carbon skeletons of LMC and GDMHAC are responsible for the signals between 97.77 and 16.14 ppm, whereas the carbon atoms in the salicylidene-imidazolium segment are responsible for the signals between 154.87 and 109.65 ppm. In addition, the two distinctive signals at 177.07 ppm and 160.72 ppm are those of the carbon atoms that are bound to the oxygen and nitrogen in the phenol and azomethine, respectively.

### 2.3. Morphological Characterization

**Figure 3:**
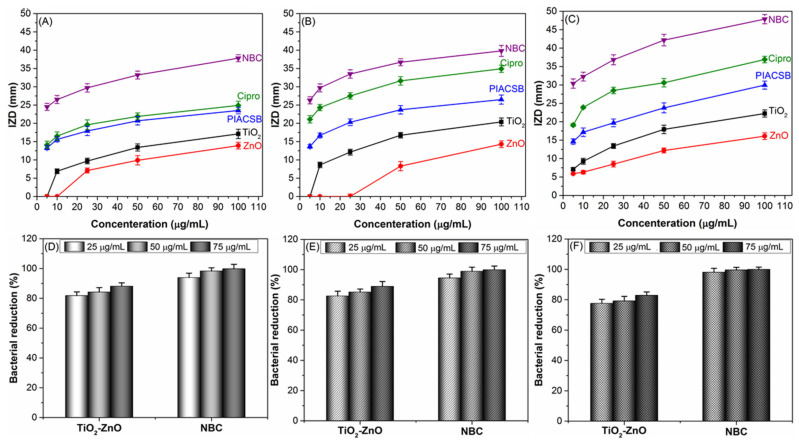
(**A**–**C**) Graph for the antibacterial inhibition zone diameters (IZD, mm) of the new NBC against (**A**) *S. aureus*, (**B**) *E. coli*, and (**C**) *P. aeruginosa*, as compared to its native constituents (PIACSB, ZnO, and TiO_2_) and a clinical control. (**D**–**F**) Impacts of the NBC on the colonization of (**D**) *S. aureus*, (**E**) *E. coli*, and (**F**) *P. aeruginosa* in their cultures using CFU assay.

SEM micrographs of the PIACSB and its ternary NBC (Figure 2D,E) were used to preliminarily inspect their surface morphologies. As can be seen in Figure 2D, the ASCSB’s surface has a rough and wrinkled appearance. The SEM micrograph of the ternary NBC (ZnO-PIACSB-TiO_2_) (Figure 2E), in contrast, showed a smooth surface with minute spherical and semi-spherical particles immobilized in its matrix. These results demonstrate that the metallic TiO_2_-ZnO nanocomposite successfully adheres to the surface of the PIACSB. Additionally, the TEM nanograph of the NBC (Figure 2F) provides a more in-depth look into its morphological aspects. The TEM nanograph of PIACSB-coated TiO_2_-ZnO nanocomposite (NBC) shows that the PIACSB forms a single-layered protective shell around the TiO_2_-ZnO that reduces the aggregation of nanoparticles and allows them to remain dispersed in a homogenous mixture. Further, TiO_2_-ZnO had a predominantly spherical shape, with a mean particle diameter (MPD) of 54.92 nm (see Figure 3G) and a low degree of polydispersity (PDI = 0.17), indicating that the size distribution of the TiO_2_-ZnO was almost evenly distributed.

### 2.4. Antimicrobial Activity Evaluation

Food spoilage is a major concern for both food producers and consumers. In most foods, the surface growth of microorganisms is the major shelf-life limiting factor. Microorganisms are responsible for the spoilage of food products, leading to significant economic losses and a considerable threat to public health. The development of microorganisms, such as bacteria, fungi, and yeasts, results in microbial spoilage, which lowers the quality of food products by producing unpleasant odors, off-flavors, and texture changes. Therefore, inhibiting the growth of microorganisms on the surface of food products is a major challenge for the food industry [39]. Despite the fact that various preservation techniques, such as pasteurization, sterilization, and refrigeration, have been developed to control microbial growth and improve the shelf life of food products, the development of antimicrobial food packaging offers a promising solution to the challenges of food spoilage. This innovative technology is efficient in preventing the growth of harmful microorganisms, thereby extending the shelf-life of food products and reducing food waste. It not only ensures the safety and quality of food but also offers a sustainable approach to food preservation [40,41].

#### 2.4.1. Antibacterial Study

*S. aureus* and *E. coli* are among the most common bacteria that cause food spoilage. These microorganisms thrive in various food products, including dairy, meat, and vegetables, and can lead to serious health risks. Moreover, *Pseudomonas* species have been found to be the primary cause of spoilage in dairy products, such as milk, cheese, and yogurt. These bacteria can grow at low temperatures and are known for their ability to produce enzymes that break down proteins and fats, causing off-flavors, off-odors, and curdling in dairy products [41]. Therefore, it is essential to practice proper food handling and packaging techniques to prevent contamination and spoilage. In this context, the antibacterial performance of the NBC and its native ingredients (PIACSB, ZnO, and TiO_2_) was evaluated using agar well-diffusion (AWD) and colony-forming unit (CFU) methods against *S. aureus*, *E. coli*, and *P. aeruginosa*. The findings of the AWD assay (see Figure 3A–C) revealed that the ternary NBC had exceptional antibacterial activity that acted against all tested bacterial strains, as compared to their native components (PIACSB, ZnO, and TiO_2_). Overall, the AWD experiment showed that Gram-negative (G^−^) bacteria (*E. coli* and *P. aeruginosa*) were more susceptible to the nanocomposite than Gram-positive (G^+^) bacteria (*S. aureus*). For instance, the antibacterial inhibition zone diameters (IZD) for ZnO-PIACSB-TiO_2_ dispersion (100 μg/mL) against the different bacterial strains, *S. aureus* (Figure 3A), *E. coli* (Figure 3B), and *P. aeruginosa* (Figure 3C), were measured as 38.11 ± 1.56 mm, 39.81 ± 1.49 mm, and 47.84 ± 1.26 mm, respectively. These IZD values verified that *P. aeruginosa* is the most susceptible to the NBC film treatment.

**Figure 4:**
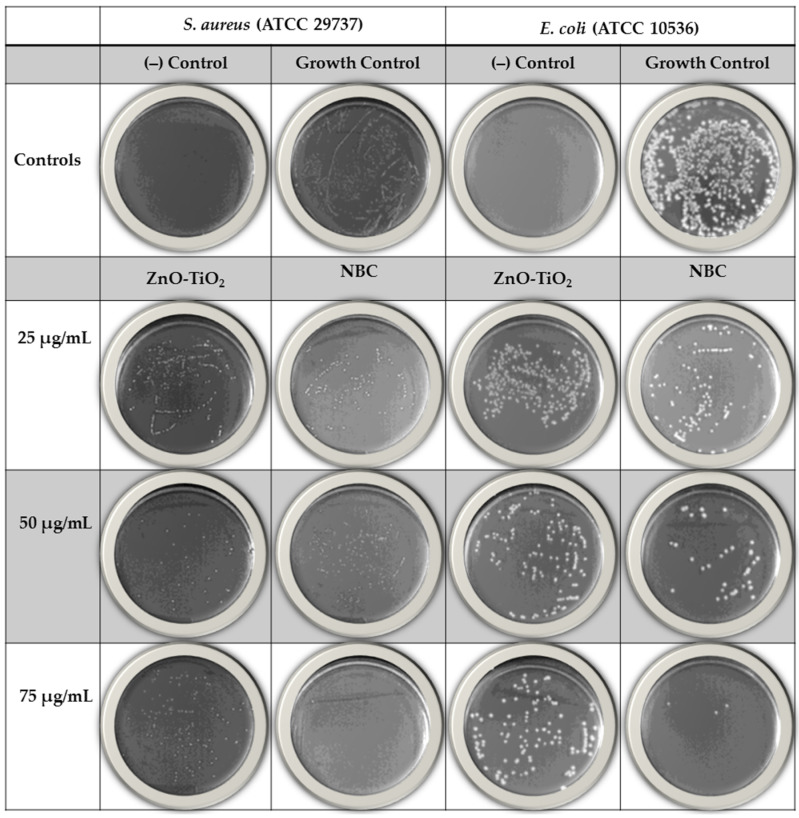
Photographs of the changes in bacterial colonies after treatment with the uncoated nanocomposite (ZnO-TiO_2_) and PIACSB-coated nanocomposite (NBC, ZnO-PIACSB-TiO_2_) as compared to negative and positive controls.

According to the outcomes of the AWD experiments, the hybrid metallic oxide nanoparticle-based NBC outperformed its native constituents (PIACSB, ZnO, and TiO_2_) in terms of bactericidal efficacy. Further antibacterial tests against the tested bacterial strains were therefore carried out on this nanocomposite and its bar nanocomposites (TiO_2_-ZnO). To that end, the impacts of serial concentrations of these materials (12.5 μg/mL to 50 μg/mL) on bacterial cultures of *S. aureus*, *E. coli,* and *P. aeruginosa* with bacterial concentrations of 1.9 × 10^6^, 2.1 × 10^6^, and 1.8 × 10^6^ CFU/mL, respectively, were investigated using the CFU assay. The results shown in Figure 3D–F and Figure 4 provide evidence that the use of the nanocomposite treatment has a significant impact (*p* < 0.05) on reducing bacterial concentrations in cultures, with a pathogen- and dose-dependent performance. For example, the colonization of *S. aureus*, *E. coli*, and *P. aeruginosa* declined by 93.8%, 94.1%, and 98.1%, respectively, in their cultures after treatment with 12.5 μg/mL of the nanocomposite. In contrast, when the nanocomposite treatment dose was raised to 50 μg/mL the bacterial populations of *S. aureus*, *E. coli*, and *P. aeruginosa* decreased to reach minimum values of 1.2%, 0.6%, and 0.1%, respectively.

The minimum inhibitory concentration (MIC) and minimum bactericidal concentration (MBC) values for the newly synthesized materials were determined using the broth dilution (BD) assay, at concentrations ranging from 0.05 to 500 μg/mL, against the tested bacterial strains. When compared to its native constituents (PIACSB, ZnO, and TiO_2_) and an uncoated nanocomposite (ZnO-TiO_2_), the MIC and MBC values of the ternary nanobiocomposite (ZnO-PIACSB-TiO_2_) against all examined bacterial species are much higher (Table 1). Furthermore, the MIC and MBC results showed that each nanocomposite was more effective against G^−^ bacteria (*E. coli* and *P. aeruginosa*) than against G^+^ bacteria (*S. aureus*), with MIC values ranging from 0.15 to 15.25 μg/mL for the former and from 0.34 to 21.75 μg/mL for the latter. Moreover, the MBC/MIC ratio is a critical measure used to determine the susceptibility or resistance of bacteria to various antibiotics. This test has been widely used to investigate the tolerance levels of bacteria and their responses to different treatments. According to Gonzalez et al., an MBC/MIC ratio (tolerance level) of ≤4 indicates that the antibiotic is bactericidal, meaning that it can kill bacteria effectively. On the other hand, an MBC/MIC ratio (tolerance level) of >4 indicates that the antibiotic is only bacteriostatic, meaning that it can only inhibit bacterial growth [42]. All tested bacterial strains had a tolerance level of <4 toward the ZnO-TiO_2_ and ZnO-PIACSB-TiO_2_ nanocomposites, indicating that they were effective bactericidal agents. Overall, the new nanocomposite (ZnO-PIACSB-TiO_2_) offers a promising new strategy for combating the rise of antibiotic resistance and could potentially be used as a supplement or alternative to traditional antibiotics.

#### 2.4.2. Antifungal Study

*A. flavus* is a fungus that is known to produce toxic compounds called aflatoxins, which can contaminate food and feedstuffs and pose a serious threat to human and animal health. In recent years, there has been an increasing focus on developing safe and effective strategies for controlling *A. flavus* growth and preventing aflatoxin contamination [43]. One promising approach is the use of hybrid nanocomposites, which have been shown to possess potent antifungal properties [43]. In this context, the antifungal activities of the bar hybrid nanocomposite (ZnO-TiO_2_) and PIACSB-coated ternary nanobiocomposite (ZnO-PIACSB-TiO_2_) were evaluated against *A. flavus* at serial concentrations in the range of 0–300 μg/mL, in comparison with their native constituents (PIACSB, ZnO, and TiO_2_). Table 1 shows that the hybrid ZnO-TiO_2_ nanocomposite has greater antifungal activity against *A. flavus* than its precursor nanoparticles (ZnO and TiO_2_) due to its high specific surface area (see Table S1, Supplementary Materials). The nanocomposite’s antifungal efficacy was traced to its propensity to produce reactive oxygen species (ROS), which are toxic to microorganisms. In addition, a higher specific surface area boosts the chances of chemical reactions and the generation of reactive oxygen species at the surface [44]. Intriguingly, the antifungal activity of the hybrid nanocomposite against *A. flavus* is greatly improved after being coated with a PIACSB film. This could be attributed to the amphiphilic nature of the PIACSB that allows for better interaction with the fungal cell membrane, leading to the disruption of the membrane and ultimately the death of the fungal cell. Additionally, the chitosan component of the PIACSB has been shown to have inherent antifungal properties, further enhancing its efficacy against *A. flavus* [45]. Notably, ZnO-TiO_2_ and ZnO-PIACSB-TiO_2_ both had much lower MIC values (38.55 and 24.85 μg/mL) against *A. flavus* than their respective native precursors (PIACSB, ZnO, and TiO_2_). On the other hand, the minimum fungicide concentrations (MFC) for ZnO-TiO_2_ and ZnO-PIACSB-TiO_2_ were 77.10 and 49.70 μg/mL, respectively. Overall, the fungicide activity of the PIACSB-coated ZnO-PIACSB-TiO_2_ hybrid nanobiocomposite was greater than that of the bare ZnO-TiO_2_ nanocomposite.

### 2.5. Antibiofilm Activity

**Figure 5:**
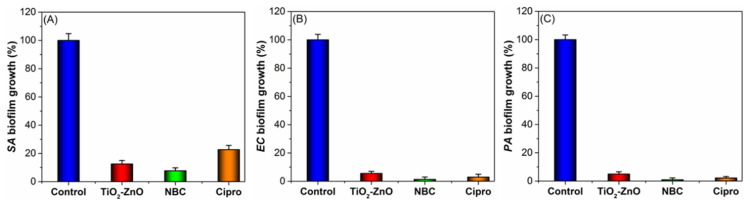
(**A**) *S. aureus*, (**B**) *E. coli*, and (**C**) *P. aeruginosa* biofilm inhibition by RNBCs, compared to Cipro and deionized water (growth control). Crystal violet staining protocol was used to quantify biofilm development.

Bacterial biofilms in the food industry have been a growing concern in recent years. These colonies of bacteria can form on various surfaces in food processing facilities, leading to the potential contamination of food products and a risk to public health. Thus, prevention and control strategies for bacterial biofilms are crucial to ensure the safety and quality of food products. In this context, we investigated the ability of ZnO-TiO_2_ nanocomposites to hinder the in vitro growth of *S. aureus*, *E. coli*, and *P. aeruginosa* biofilms on polystyrene surfaces. The findings demonstrated that both nanocomposites were strongly effective in reducing the viability of bacterial biofilms when compared to the growth controls. Generally, the antibiofilm activities of the ZnO-TiO_2_ nanocomposites were shown to be more effective against enterococcal and *P. aeruginosa* biofilms than against staphylococcal biofilms. Particularly, the PIACSB-coated hybrid nanobiocomposite (ZnO-PIACSB-TiO_2_) exhibited very strong antibiofilm effects against *S. aureus* (only 8.7% ± 1.9 biofilm growth), *E. coli* (only 1.4% ± 1.1 biofilm growth) and, *P. aeruginosa* (only 0.85% ± 1.3 biofilm growth), which were superior to those of the positive control (Cipro) (22.3% ± 2.1, 2.9% ± 1.7, and 2.1% ± 1.7 for the same sequence of bacterial biofilms, respectively) (see Figure 5).

### 2.6. Proposed Molecular Mechanisms of Antimicrobial and Antibiofilm for Action of NBC

**Figure 6:**
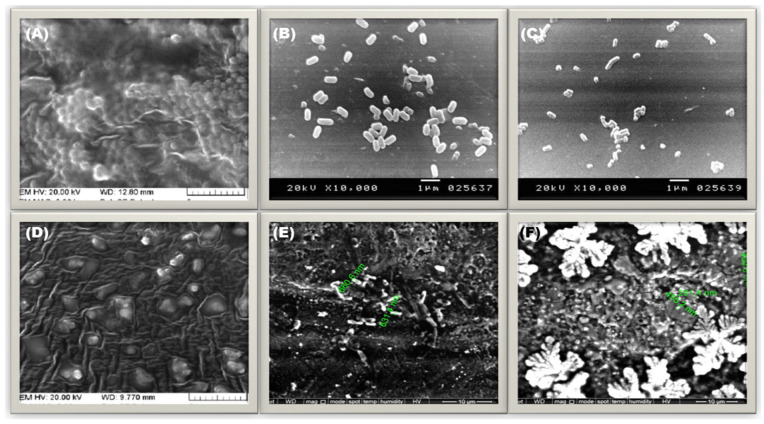
(**A**–**C**) SEM micrographs of the untreated (control) bacterial cell cultures (**A**) *S. aureus*, (**B**) *E. coli*, and (**C**) *P. aeruginosa*. (**D**–**F**) SEM micrographs of the NBC-treated (control) bacterial cell cultures (**D**) *S. aureus*, (**E**) *E. coli*, and (**F**) *P. aeruginosa*. The scale for pictures (**A**–**D**) is 1 μm, while for pictures (**E**,**F**), it is 10 μm.

The superior antibacterial activity of ZnO-PIACSB-TiO_2_ nanocomposite could be attributed to the physicochemical properties and synergistic effects of its ingredients. The high nanocomposite surface area allows for greater interaction between the bacterial cells and the nanocomposite, while the zinc and titanium ions can disrupt the bacterial cell wall and interfere with its metabolic processes. Secondly, the ZnO-TiO_2_ nanocomposite’s antibacterial activity is attributed to the release of reactive oxygen species (ROS), which can damage the bacterial membrane and inhibit its growth. The acidic environment created by the nanocomposite can trigger the release of hydrogen ions, which can further damage the bacterial membrane and increase its susceptibility to the nanocomposite’s antibacterial effects [46,47]. On the other hand, there are a variety of reasons behind the excellent antibacterial action of the PIACSB film. One of the reasons is that its positively charged surface interacts with the negatively charged membranes of microorganisms, causing the leaking of proteinaceous and other internal contents of the pathogen [48,49]. In particular, the chitosan matrix has been shown to be more efficient against G^−^ bacteria than G^+^ bacteria [50]; nonetheless, chitosan on the surface of the G^+^ cell can form a polymeric wall that restricts nutrients from entering the cell [51], ultimately causing cell death. Further, the amphiphilic chitosan disrupts the bacterial cell membrane, leading to the leakage of intracellular contents and ultimately cell death. This membrane-disrupting effect of amphiphilic chitosan is attributed to its ability to interact with the lipid bilayer of the bacterial cell membrane. The hydrophobic alkyl chains of the amphiphilic chitosan interact with the hydrophobic tails of the membrane lipids, while the hydrophilic chitosan backbone interacts with the hydrophilic head groups of the lipids. This interaction leads to the formation of pores in the membrane, which results in the leakage of intracellular contents and eventually leads to cell death (see Figure 6). Finally, the presence of both hydrophilic and hydrophobic groups allows amphiphilic chitosan to self-assemble into micelles, which can enhance its solubility and bioavailability, as well as provide a means for the targeted delivery of the antimicrobial agent [52,53].

Several factors were responsible for the superior antibiofilm activity of the PIACSB-coated hybrid nanocomposite (ZnO-PIACSB-TiO_2_). Initially, the smaller size of the NBC allows it to penetrate the biofilm matrix more easily, leading to a better interaction with the bacterial cells and enhanced antibiofilm activity [54]. The synergistic antibiofilm effects the NBC ingredients (TiO_2_, ZnO, and PIACSB) through multiple mechanisms. TiO_2_NPs have been shown to exhibit antibiofilm activities due to their ability to generate reactive oxygen species (ROS). These ROS can cause damage to the bacterial cell membrane, leading to bacterial death. Additionally, TiO_2_NPs have been shown to disrupt biofilm formation by inhibiting the attachment of bacteria to surfaces and disrupting the bacterial cell wall and membrane. The nanoparticles can also induce oxidative stress and damage DNA, leading to bacterial death. Furthermore, TiO_2_NPs can interfere with quorum-sensing, a process that regulates gene expression in bacteria and plays a critical role in biofilm formation. Finally, the nanoparticles can disrupt the communication between bacterial cells, preventing them from coordinating the formation of a biofilm [55,56,57]. The superior antibiofilm activities of ZnONPs could be attributed to several factors [58]. Firstly, the unique physicochemical properties of ZnONPs enable them to interact with bacterial cells. Secondly, ZnONPs can interact with bacterial membranes and disrupt their integrity. Lastly, ZnONPs can inhibit quorum-sensing and virulence genes, which are essential for biofilm formation [59]. In addition, the superior antibiofilm activity of amphiphilic chitosan is a promising solution in the fight against microbial infections. This remarkable property can be attributed to several factors, including the ability of the PIACSB to penetrate both hydrophilic and hydrophobic components of the biofilm matrix, allowing the ZnO-TiO_2_ nanocomposite to enter the bacterial cells more efficiently, inducing further bacterial biofilm destruction. Further, the high surface charge density enables it to disrupt cell membranes and the antiadhesive properties prevents bacterial attachment and colonization [60]. Based on these findings, it can be concluded that the new hybrid NBC is a promising material for the development of biofilm inhibition strategies for the prevention of biofilm-associated infections.

### 2.7. Antimicrobial NBC vs. Previous TiO_2_-ZnO-Based Nanocomposites

According to a previous study, the values of MIC and MBC reported for various TiO_2_-ZnO nanocomposites were in the ranges of MIC/MBC 15.8/15.8–1000/2000 μg/mL, 31.2/31.2–2000/3000 μg/mL, and 9.6/9.6–31.2/31.2 μg/mL against *S. aureus*, *E. coli*, and *P. aeruginosa*, respectively (see Table S2, Supplementary Materials) [61,62,63,64]. However, the newly synthesized PIACSB-coated TiO_2_-ZnO nanocomposite is particularly exciting due to its amazing antimicrobial activity (MIC/MBC→0.34/0.68 μg/mL, 0.20/0.40 μg/mL, and 0.15/0.30 μg/mL against *S. aureus*, *E. coli*, and *P. aeruginosa*, respectively) and unique properties. PIACSB is a biocompatible and biodegradable polysaccharide that has been shown to enhance the antimicrobial activity of nanoparticles. When combined with TiO_2_-ZnO nanocomposites, it forms a highly effective antibacterial agent that could be used in a wide range of medical applications. The PIACSB coating also helps to improve the stability and biocompatibility of the nanocomposites, making them safer and more effective for use in both in vitro and in vivo studies. Overall, the promising results of this study suggest that PIACSB-coated TiO_2_-ZnO nanocomposites could offer a new and effective way to combat bacterial infections and improve human health.

### 2.8. Cytotoxicity

The cytotoxic effects of two types of nanocomposites, bar hybrid (ZnO-TiO_2_) and PI-ACSB-coated hybrid (ZnO-PIACSB-TiO_2_), were assessed in vitro using the MTT assay on mouse (L929) and human skin (HSF) fibroblast cell lines. Concentrations ranging from 3.125 to 200 µg/mL were used. The PIACSB-coated nanocomposite has lower cytotoxicity against fibroblasts compared to the uncoated nanocomposite (ZnO-TiO_2_), as shown in Figure S8 (Supplementary Materials). At a 100 µg/mL dose, application of the PIACSB-coated nanocomposite resulted in 36.1% and 29.4% toxicity against L929 and HSF, respectively, while the application of the uncoated nanocomposite resulted in 74.3% and 69.9% toxicity against L929 and HSF, respectively. Meanwhile, excellent nanocomposite biocompatibility is demonstrated by an IC_50_ value for the PIACSB-coated nanocomposite against both cell lines, which is substantially high (IC_50_ 135–143 µg/mL). According to the Food and Drug Administration (FDA), biocompatibility refers to the ability of a material to perform its intended function without causing adverse effects on living tissue. In this regard, the IC_50_ values of the nanocomposite against both fibroblasts have been shown to be within the 135–143 µg/mL range, which is significantly higher than that of the MIC doses (0.24–24.85 µg/mL) against all tested microbes. Therefore, this indicates that the nanocomposite has biocompatibility according to ISO 10993-5 “Biological evaluation of medical devices-part 5-Tests for in vitro cytotoxicity” [65], which is essential for antimicrobial food packaging applications. The reason for this higher biocompatibility was attributed to the presence of biocompatible amphiphilic chitosan film in the NBC [66]. These results suggest that the nanocomposite has better biocompatibility, making it a promising candidate for biomedical and food packaging applications; nevertheless, additional future research will be performed to comprehensively comprehend the intricate biocompatibility and cytotoxicity impacts of this nanocomposite before its implementation in the food packaging sector.

## 3. Materials and Methods

The Supplementary Materials section includes comprehensive information on the properties and sources of all chemicals, solvents, and other materials utilized in this study. The starting materials, glycidyldimethylhexadecyl ammonium chloride (GDMHAC), lower molecular weight chitosan (LMC), ZnONPs, and TiO_2_NPs, were obtained from our previous work. In addition, the preparation and characterization of the functionalized imidazolium ionic liquid (2), polyimidazolium-supported chitosan Schiff base (PICSB), and PIACSB are described in the Supplementary Materials section. The structural characteristics of the novel materials were inspected using microanalytical and spectral techniques (FTIR, UV-Vis, NMR, and ESI-MS), as well as physical measurements. The Supplementary Materials section also included specifics on the instruments utilized to conduct these studies.

### 3.1. In Situ Preparation of Hybrid Nanobiocomposite (NBC) (ZnO-PIACSB-TiO_2_)

Figure 1 shows a detailed step-by-step methodology for preparing the starting ingredients and intended NBC. The ZnONPs were synthesized using a co-precipitation technique while preparing the TiO_2_NPs using a sol-gel method mediated by ultrasonic irradiation in a PIACSB dispersion [32,67]. The first step was to prepare stock solutions of 1.0% PIACSB, 0.3 M Ti^4+^ precursor (titanium isopropoxide, Ti(O^i^Pr)_4_), and 0.27 M Zn^2+^ precursor (zinc acetate, Zn(OAc)_2_) in 1% aqueous acetic acid/ethanol (1:1 *v*/*v*), isopropanol, and deionized water, respectively. Subsequently, the ternary mixture was concocted through the blending of 10 mL of PIACSB dispersion with 1 mL of Ti^4+^ and Zn^2+^ solutions each while being subjected to magnetic stirring. The mixture was then subjected to ultrasonic irradiation for 15 min, followed by the gradual addition of 10 mL of NaOH solution (1.355 M) (1 mL/min) under magnetic stirring. A further 20 min of ultrasonic irradiation (200 W, Bandelin, SONOPULS, 20 kHz, Berlin, Germany) was applied once the NaOH addition was finished. The obtained ternary nanocomposite (NBC) (ZnO-PIACSB-TiO_2_) was collected by centrifugation at 15 × 10^3^ rpm for 30 min, thoroughly washed in ethanol and deionized water, and finally dried at 150 °C for 2 h.

### 3.2. Antimicrobial Study

AWD and CFU methods were used to evaluate the new materials for their antimicrobial activities against the most prevalent foodborne pathogens *Staphylococcus aureus* (*S. aureus*, ATCC 29737), *Escherichia coli* (*E. coli*, ATCC 10536), *Pseudomonas aeruginosa* (*P. aeruginosa*, ATCC 27853), and *Aspergillus flavus* (*A. flavus*, ATCC 46283), which were provided by VACSERA, Egypt, in accordance with our previously published antimicrobial test procedures [54,68], which are briefly described in the Supplementary Materials section. The antibiotic ciprofloxacin (Cipro) served as a reference standard.

### 3.3. Antibiofilm Study

Our earlier research [54] was used to guide the antibiofilm assay of the novel nanocomposites. Briefly, an aqueous sample solution was applied to 96-well flat-bottom microtiter plates (10 μg/mL, 30 μL/well) and allowed to dry at 37 °C for 24 h. A negative control was provided by deionized water (DIW). After overnight cultivation of the bacterial species at 37 °C on tryptic soy agar (TSA), a few colonies from each bacterium were suspended in 2% glucose-containing tryptic soy broth (TSB). The suspension was subjected to a 60 s vortexing; then, its OD_600_ (nm) value was set to 0.08, equivalent to a concentration of around 10^7^ CFU/mL. Two hundred microliters of the diluted bacterial suspension was then applied to each pre-coated well; wells without inoculated TSB medium served as growth controls. Finally, after 24 h of incubation at 37 °C, the biofilm growth was quantified by staining the plates with crystal violet and measuring the absorbance at 600 nm on a microplate reader.

### 3.4. In Vitro Cytotoxicity Study

The cytotoxicity performances of the novel pharmaceutical materials were studied against two fibroblast cell lines, mouse (L929) and human skin (HSF) fibroblasts. These investigations were conducted using the MTT test in accordance with our prior research [30] and are briefly described in the Supplementary Materials section.

### 3.5. Statistics

In this work, the data obtained were subjected to graphical presentations, statistical analyses, and mathematical treatments using the software packages of OriginPro 9.1.32 and SPSS v17 programs, which ensured the accuracy and reliability of the results. In addition, the *p*-value was used as a measure of statistical significance. If the *p*-value of an outcome was less than 0.05, it was regarded as statistically significant.

## 4. Conclusions

This study presents a new environmentally friendly method for producing a multifunctional nanocomposite (NBC) (ZnO-PIACSB-TiO_2_) with excellent antimicrobial and antibiofilm properties. The NBC is created by co-encapsulating and capping a mixture of ZnONPs and TiO_2_NPs. Initially, a series of consecutive physical processes and chemical reactions (deproteinization, demineralization, deacetylation, partial degradation, Schiff base condensation with SIIL, and eventually *O*-quaternization by GDMHAC) were used to upgrade the shrimp shells to the PIACSB. Subsequently, the PIACSB was utilized as both an encapsulating and coating agent in the in situ synthesis of a hybrid NBC (ZnO-PIACSB-TiO_2_) through the simultaneous in situ formation of ZnONPs and TiO_2_NPs. The new materials were physically, structurally, and mophologically characterized based on the “ζ” potential measurements, thermal, elemental analysis, spectral, and microscopic techniques. The new hybrid NBC has the significant potential to synergistically inhibit the colonization of foodborne pathogens (bacteria and fungi) and prevent the development of bacterial biofilms. This study found that the PIACSB-coated hybrid nanocomposite (ZnO-PIACSB-TiO_2_) had higher antimicrobial indices (MIC/MBC, tolerance, and MIC/MFC) compared to the uncoated hybrid nanocomposite (ZnO-TiO_2_), indicating its superior effectiveness in inhibiting bacterial and fungal growth. Its activity surpassed that of the clinical antimicrobial medication Cipro. Meanwhile, acceptable nanocomposite biocompatibility is demonstrated by IC_50_ values for the PIACSB-coated nanocomposite acting against L929 and HSF cell lines that are substantially greater (IC_50_ 135–143 µg/mL) than those of an uncoated one (IC_50_ = 19.36 ± 2.04–23.48 ± 1.56 µg/mL). These results suggest that the new PIACSB-coated nanobiocomposite film may provide promising multifunctional food packaging additives, meeting the needs of customers for safe and eco-friendly food items with superior antimicrobial and antibiofilm properties.

## Figures and Tables

**Table 1:** Antimicrobial indices for prepared materials: MIC/MBC (μg/mL), tolerance, and MIC/MFC (μg/mL) against different tested microbial strains, as well as their cytotoxicity indices: IC_50_ (μg/mL) against the two fibroblast cell line (HSF and L929).

Parameter	IC_50_ (μg/mL) HSF Cells	IC_50_ (μg/mL) L929 Cells	MIC/MBC (μg/mL)			Tolerance ^a^ *SA*/*EC*/*PA*	MIC/MFC (μg/mL)
Sample			*S. aureus*	*E. coli*	*P. aeruginosa*		*A. flavus*
TiO_2_	14.97 ± 0.31	13.45 ± 0.25	250/250	225/225	200/200	1/1/1	76.25/152.5
ZnO	67.28 ± 2.25	53.63 ± 1.08	300/600	250/500	225/225	2/2/1	155/310
PIACSB	>250	>250	62.5/125	62.5/125	56.25/112.5	2/2/2	83.75/83.75
TiO_2_-ZnO	23.48 ± 1.56	19.36 ± 2.04	21.75/21.75	15.25/30.50	9.25/9.25	1/2/1	38.55/77.10
NBC	143	135	0.34/0.68	0.20/0.40	0.15/0.30	2/2/2	24.85/49.70
Cipro	NA	NA	0.75/3.01	0.5/1.0	0.45/0.91	4/2/2	NA

^a^ Tolerance = MBC/MIC.

## Data Availability

Not applicable.

## Supplementary Materials

The following supporting information can be downloaded at: https://www.mdpi.com/article/10.3390/antibiotics12071110/s1. Supplementary File S1: 1. Materials and instrumentation; 2. 5-chloromethyl-2-hydroxybenzaldehyde (1); 3. Synthesis of 5-(1,2-dimethylimidazolium) salicylaldehyde chloride (SIIL, 2); 4. Extraction of chitin from shrimp shells; 5. Preparation of chitosan; 6. Preparation of low molecular weight chitosan (LMC); 7. Synthesis of polyimidazolium-supported chitosan Schiff base (PICSB); 8. Synthesis of polyimidazolium-supported amphiphilic chitosan Schiff base (PIACSB); 9. Antimicrobial activity; 10. In vitro cytotoxicity activity. Figure S1: EDS spectrum of PIACSB; Figure S2: EDS spectrum of ZnO-PIACSB-TiO2 (NBC); Figure S3: FTIR of SIIL (2); Figure S4: FTIR of LMC; Figure S5: FTIR of PICSB; Figure S6: 1HNMR (200 MHz) of PIACSB in D2O; Figure S7: 13CNMR (125 MHz) of PIACSB in D2O. Figure S8: Inhibitory effects of serial doses (3.125–200 μg/mL) of (A) ZnO-TiO2 and (B) ZnO-PIACSB-TiO2 on the proliferative activities of L929 and HSF cells; Table S1: Molecular structure indices (Mav, EA, DD, and QD) of new stabilizers (SIIL and PIACSB) and TiO2-ZnO nanocomposites; Table S2: Antimicrobial NBC vs. previous TiO_2_-ZnO-based nanocomposites
