# Correction: Abd El-Fattah et al. Immobilization of ZnO-TiO_2_ Nanocomposite into Polyimidazolium Amphiphilic Chitosan Film, Targeting Improving Its Antimicrobial and Antibiofilm Applications. *Antibiotics* 2023, *12*, 1110

**DOI:** 10.3390/antibiotics13090836

**Published:** 2024-09-02

**Authors:** Wesam Abd El-Fattah, Mohammad Y. Alfaifi, Jafar Alkabli, Heba A. Ramadan, Ali A. Shati, Serag Eldin I. Elbehairi, Reda F. M. Elshaarawy, Islam Kamal, Moustafa M. Saleh

**Affiliations:** 1 Chemistry Department, College of Science, IMSIU (Imam Mohammad Ibn Saud Islamic University), P.O. Box 5701, Riyadh 11432, Saudi Arabia; wabdulfatah@imamu.edu.sa; 2 Department of Chemistry, Faculty of Science, Port Said University, Port Said 42521, Egypt; 3 Biology Department, Faculty of Science, King Khalid University, Abha 61413, Saudi Arabia; aaalshati@kku.edu.sa (A.A.S.); serag@kku.edu.sa (S.E.I.E.); 4 Department of Chemistry, College of Sciences and Arts—Alkamil, University of Jeddah, Jeddah 23218, Saudi Arabia; jabdalsamad@uj.edu.sa; 5 Department of Microbiology and Immunology, Faculty of Pharmacy, Delta University for Science and Technology, Mansoura 11152, Egypt; hebaaa.aadel@gmail.com; 6 Department of Chemistry, Faculty of Science, Suez University, Suez 43533, Egypt; 7 Institute for Inorganic Chemistry and Structural Chemistry, Düsseldorf University, 40225 Düsseldorf, Germany; 8 Department of Pharmaceutics, Faculty of Pharmacy, Port Said University, Port Said 42526, Egypt; islamkamal@pharm.psu.edu.eg; 9 Microbiology and Immunology Department, Faculty of Pharmacy, Port Said University, Port Said 42526, Egypt; mostafa.mohamed@pharm.psu.edu.eg

## Error in Figure

In the original publication [1], there was a mistake in “Figure 4” as published. There was an accidental mistake made during the revision process by duplicating some images in the right hand column in Figure 4. The corrected “Figure 4” appears below. The authors state that the scientific conclusions are unaffected. This correction was approved by the Academic Editor. The original publication has also been updated.

## Figures and Tables

**Figure 4:**
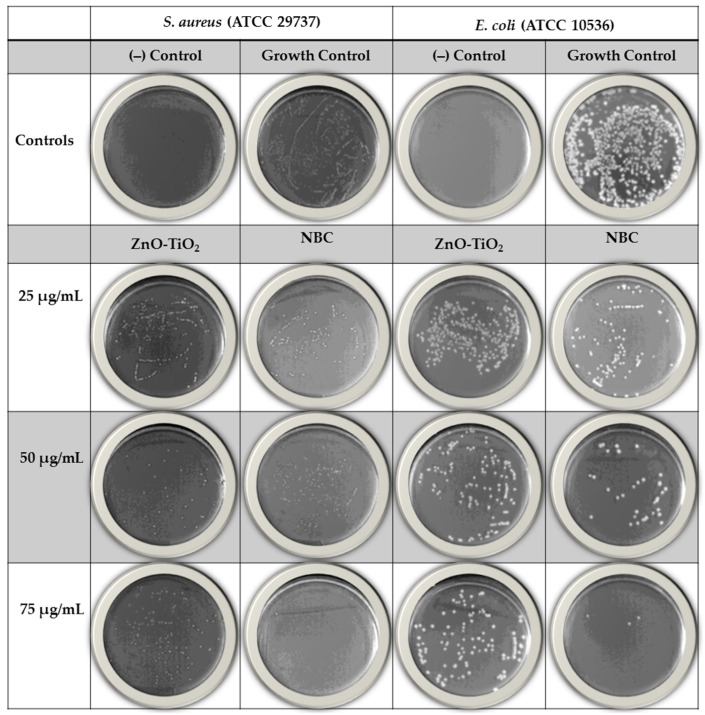
Photographs of the changes in bacterial colonies after treatment with the uncoated nanocomposite (ZnO-TiO_2_) and PIACSB-coated nanocomposite (NBC, ZnO-PIACSB-TiO_2_) as compared to negative and positive controls.
