# Supplementary material for: Immobilization of ZnO-TiO2 Nanocomposite into Polyimidazolium Amphiphilic Chitosan Film, Targeting Improving Its Antimicrobial and Antibiofilm Applications
Source: Antibiotics (Basel). 2023 Jun 27;12(7):1110. doi: 10.3390/antibiotics12071110 (PMC10376717; doi:10.3390/antibiotics12071110)
Supplement: Supplementary file 1 [file antibiotics-12-01110-s001.zip › antibiotics-2435319-supplementary.pdf]

Immobilization of $ZnO-TiO_2$ nanocomposite into polyimidazolium amphiphilic chitosan film; targeting improving its safe antimicrobial and anti-biofilm applications

Wesam Abd El-Fattah 1,2, Mohammad Y. Alfaifi 3,* , Jafar Alkabli 4, Heba A. Ramadan 5, Ali A. Shati 3, Serag Eldin I. Elbehairi 3 , Reda F. M. Elshaarawy 6,7,* , Islam Kamal 8 and Moustafa M. Saleh 9

1 Chemistry Department, College of Science, IMSIU (Imam Mohammad Ibn Saud Islamic University), P.O. Box 5701, Riyadh 11432, Saudi Arabia; wabdulfatah@imamu.edu.sa
2 Department of Chemistry, Faculty of Science, Port Said University, Port Said 42521, Egypt
3 Biology Department, Faculty of Science, King Khalid University, Abha 61413, Saudi Arabia; aaalshati@kku.edu.sa (A.A.S.); serag@kku.edu.sa (S.E.I.E.)
4 Department of Chemistry, College of Sciences and Arts—Alkamil, University of Jeddah, Jeddah 23218, Saudi Arabia; jabdalsamad@uj.edu.sa
5 Department ofMicrobiology and Immunology, Faculty of Pharmacy, Delta University for Science and Technology, Mansoura 11152, Egypt; hebaaa.aadel@gmail.com
6 Department of Chemistry, Faculty of Science, Suez University, Suez 43533, Egypt
7 Institute for Inorganic Chemistry and Structural Chemistry, Düsseldorf University, 40225 Düsseldorf, Germany
8 Department of Pharmaceutics, Faculty of Pharmacy, Port Said University, Port Said 42526, Egypt; islamkamal@pharm.psu.edu.eg
9 Microbiology and Immunology Department, Faculty of Pharmacy, Port Said University, Port Said 42526, Egypt; mostafa.mohamed@pharm.psu.edu.eg
* Correspondence: alfaifi@kku.edu.sa (M.Y.A.); reda.elshaarawy@suezuniv.edu.eg (R.F.M.E.)

Contents:

1- **Materials and instrumentation**
2- **Synthesis**
3- **Figures**
4- **Tables**

1. Materials and instrumentation

Chemicals were obtained from the following suppliers and used without further purification: toluene, cumene, tert-butylbenzene, 1,2-dimethylimidazole, $NaBH_4$, and anhydrous $MgCl_2$ (Sigma–Aldrich); Zinc iodide (Acros).

Melting points were measured using a BÜCHI Melting point B-540 apparatus; all melting points were measured in open glass capillaries and are uncorrected. Elemental analyses for C, H, N, were performed with a Perkin–Elmer 263 elemental analyzer. FT-IR spectra were recorded on a BRUKER Tensor-37 FT-IR spectrophotometer in the range 400–4000 cm^{-1} as KBr disc in the 4000-550 cm^{-1} region with 2 cm^{-1} resolution or with an ATR (attenuated total reflection) unit (Platinum ATR-QL, diamond). For signal intensities the following abbreviations were used: br (broad), sh (sharp), w (weak), m (medium), s (strong), vs (very strong). UV/Vis spectra were measured at 25 °C in ethanol (10^{-5} mol/L) on a Shimadzu UV-2450 spectrophotometer using quartz cuvettes (1 cm). NMR-spectra were obtained with a Bruker Avance DRX200 (200 MHz for ^1H) or Bruker Avance DRX500 (125 and 470 MHz for ^{13}C and ^{19}F respectively) spectrometer with calibration to the residual proton solvent signal in DMSO-d_6 (^1H NMR: 2.52 ppm, ^{13}C NMR: 39.5 ppm), $CDCl_3$ (^1H NMR: 7.26 ppm, ^{13}C NMR: 77.16 ppm) against TMS ($\delta = 0.00$ ppm) for ^1H and ^{13}C, 85% phosphoric acid ($\delta = 0.00$ ppm) for ^{31}P and $CFCl_3$ ($\delta = 0.00$ ppm) for ^{19}F NMR. Multiplicities of the signals were specified s (singlet), d (doublet), t (triplet), q (quartet) or m (multiplet). The ESI-MS of the synthesized compounds were acquired in the linear mode for positive ions on a UHR-QTOF maXis 4G (Bruker Daltonics) and BRUKER Ultraflex MALDI-TOF instrument equipped with a 337 nm nitrogen laser pulsing at a repetition rate of 10 Hz. The 2+ charge assignment of ions in HR-ESI-MS was confirmed by the m/z = 0.5 difference between the isotope peaks (x, x+1, x+2). Peaks with chlorine showed the isotope ratio $^{35/37}Cl$ = 75.8:24.2.

2. *5-chloromethyl-2-hydroxybenzaldehyde* (1)

It was synthesized from the corresponding salicylaldehyde according to the modified chloromethylation procedure. In a typical synthesis, (15.2 mmol) of salicylaldehyde was treated with para-formaldehyde (1.0 g, 33.3 mmol) and zinc chloride (0.2 g, 1.46 mmol) in 20 ml of concentrated hydrochloric acid. The mixture was vigorously stirred under HC1g atmosphere for 24-72 h at 313 K. The reaction mixture was extracted several times with diethyl ether (3x15 mL). Then the collected ether fractions were washed by 2x10 mL 5% aqueous NaHCO3 solution, 2x10 mL brine, 5x10 mL milli-Q water and dried over anhydrous MgSO4. After filtration and removal of the volatiles under reduced pressure, the obtained product was characterized and used in the next step without further purification. 5-Chloromethyl-2-hydroxybenzaldehyde was isolated as white needles (15.2 g, 62.0 % yield). FTIR (ATR, cm-1): 3240 (m, br, ν OH), 3120 (m, br, νasym CH, Ar), 3050 (m, br, νsym CH, Ph), 2876 (m, sh, ν CH2), 1659 (vs, sh, ν C=O), 1578, 1489, 1437 (s, sh, ν C=Carom + ν C-H bend), 1338 (m, sh, ν CH2), 1252 (s, sh, ν CH2Cl), 1150 (s, sh, ν HCC, Ar), 772 (s, sh, ν C–Cl). 1H NMR (200 MHz, CDCl3) δ (ppm): 11.07 (s, 1H, Ar-OH) 9.90 (s, 1H, Ar-HC=O), 7.57 (m, 2H, 2 x Ar-H), 7.00 (d, 1H, J = 8.34 Hz, Ar-H), 4.60 (s, 2H, CH2-Ar).

3. Synthesis of 5-(1,2-dimethylimidazolium) salicylaldeide chloride (SIIL, 2)

Into a three-neck round-bottom flask, 5-chloromethyl-2-hydroxybenzaldehyde (0.03 mol) was added dropwise to a solution of 1,2-dimethylimidazole (0.03 mol) in toluene (30 mL) at room temperature under stirring. The mixture was then heated under reflux in a N_2 atmosphere for about 48 h, until no starting material

was detected by thin layer chromatography (TLC). After the reaction was completed, the isolated product was washed intensively with 2 x 5 mL dry toluene, several with ether (5x10 mL), to remove the unreacted materials, and dried under vacuum to give the desired products which used for the following preparations without further purification. It was obtained as of white solid, Yield (89 %). FTIR (KBr, cm^{-1}): 3373 (m, br, $v_{(O-H)}$), 1669 (vs, sh, $v_{(C=O)}$), 1547, 1455, 1399 (s, sh, $v_{(C=CAr + C-Hbend)}$), 1274 (s, sh, v $v_{(Ar-O)}$), 1153 (s, sh, $v_{(H-C=C + H-C=N)bend}$, Im). ^{1}H NMR (200 MHz, DMSO-d_6) δ (ppm): 10.80 (s, 1 H, Ar-O**H**), 10.33 (s, 1 H, Ar-**H**C=O), 7.84 (d, *J* = 1.76 Hz, 1 H, N(1)CHC**H**N(3)), 7.67 (d, *J* = 2.01 Hz, 2 H, 2 x Ar-**H**), 7.55 (d, *J* = 1.69 Hz, 1 H, N(1)C**H**CHN(3)), 7.41 (m, 3H, 3 x Ar-**H**), 5.38 (s, 2H, N(3)-C**H₂**-Ar), 3.86 (s, 3 H, N(1)-C**H₃**), 2.60 (s, 3H, C(2)-C**H₃**).^{13}C NMR (125 MHz, DMSO-d_6) δ (ppm): 197.23, 160.18, 144.71, 137.45, 136.23, 131.03, 127.72, 122.80, 121.91, 120.13, 51.34, 35.39 and 22.16. MALDI-TOF MS, *m/z*: 231.21 [$C_{13}H_{15}N_2O_2$, M - Cl^-]$^+$.

4. Extraction of chitin from shrimp shells

Fresh samples of shrimp shells were obtained from the local market of Suez. The samples were washed thoroughly with water and then dried for 24 h. All chemicals used are analytical grade chemicals. The collected shrimp wastes were then washed with tap water and crushed with mortar pastle. Crushed shrimp waste was kept in a polyethylene bags at ambient temperature (28±2°C) for 24 hours for partial autolysis to facilitate chemical extraction of chitosan and to improve the quality of chitosan.

The following conditions were chosen as an optimal extractive treatment: The first stage of the extraction process involves a thermomechanical treatments, where the shells are scraped free of loose tissue and washed individually in lightly saline water, then separated from cephalothoraxes, salted (5 kg of NaCl per 250 g of shell), washed thoroughly in distilled water, dried in the sun (25-30 °C) for 3 days, and finally dried in an oven at 60 ºC for 48 hr. After that, the dried shells were grinded, sieved, and the fraction below 80 μm was used hereafter. The second stage started with a demineralization process which was carried out using 0.5 M HCl solutions. Typically, 100 g of shrimp shells powder was immersed in 1000 ml of 0.5 M HCl at ambient temperature (25 °C) under constant stirring for 24 h. After filtration, the residue was washed with distilled water until the pH of the rinsed water became neutral. Then the residue was subjected to deproteination, by immersing in 1000 mL of 1 M NaOH under vigorous stirring at 60 °C for 24 h. Then the proteins were removed by filtration. Distilled water was used to wash the residue to neutral. Then, the shrimp shell residue was subjected to the above procedure, starting with the thermochemical treatment, two more times. The chitin obtained still had a slight pink colour. Further decolourisation was achieved by soaking chitin in 250 mL of 1% $KMnO_4$ for 1 h. followed by 250 mL of 1% oxalic acid for 2 h. The amount of 250 mL of 95% ethanol and 200 mL of absolute ethanol were sequentially used to remove ethanol-soluble substances from the obtained crude chitin and to dehydrate the chitin. Finally the chitin was dried in air at 50 °C overnight. Yield 96.34 g (96.34% based on 100 g of shrimp shell powder)

5. Preparation of chitosan

The purified chitin was deacetylated to form chitosan by treating 10 g of chitin with 100 mL 65 % NaOH under stirring at 60 °C for 72 h. After filtration, the residue was washed three times with 10 mL of hot deionized water at 60 °C. The crude chitosan (7.9 g) was obtained by drying in an air oven at 50 °C overnight. The obtained crude chitosan was purified by dissolution in 1% (v/v) aqueous acetic acid until a homogenous solution is obtained, filtered through 22 μm Whatman filter paper to remove insoluble impurities, then precipitated by titration with 1 N NaOH until pH value of 8.5, and finally washed several times with distilled water. Yield 7.5 g (92.6 % based on chitin). FTIR (KBr, cm^{-1}): 3462 (m, br, $\nu_{(O\text{-}H+\ NH_2)}$), 3101 (m, br, $\nu_{(N\text{-}H)}$), 1653 (vs, sh, $\nu_{(C=O)acetyl}$), 1568 (m, sh, amide II), 1380 (m, sh, amide III), 1069 (m, sh, $\nu_{(C\text{-}O\text{-}C)str}$), 896 (m, sh, $\nu_{(C\text{-}O\text{-}C)}$, β-glycosidic linkage). ^{1}H NMR (600 MHz, 1% $CD_3COOD/D_2O)_{60\ °C}$ δ (ppm): 5.22 (d, J = 7.2 Hz, 1H, $GlcNH_2$ residue), 4.24 (m, 2H, $GlcNH_2$ and GlcNAc residue), 4.12-3.90 (m, 3H, $GlcNH_2$ and GlcNAc residue), 3.53 (s, 1H, $GlcNH_2$ residue), 2.40 (s, 3H, NHAc). ^{13}C NMR (151 MHz, 1% $CD_3COOD/D_2O)_{60\ °C}$ δ (ppm): 177.03, 98.16, 94.35, 90.46, 83.89, 75.27, 70.73, 68.57, 62.80, 60.53, 56.28, 46.01, 32.61 and 23.54. Degree of acetylation 24.3%. Anal. Calcd. for $(C_8H_{13}NO_5)_{0.243}$ $(C_6H_{11}NO_4)_{0.757}$·H_2O (M = 189.39 g/mol) : C, 41.13; H, 7.18; N, 7.18;; Found C, 40.63; H, 7.12; N, 7.31.

6. Preparation of low molecular weight chitosan (LMC)

Chitosan solution (2%) was prepared in 1% CH_3COOH by stirring for 24 h in room temperature. A solution of H_2O_2 30% (4.4%) was then used to achieve chemical degradation of chitosan in 30 for 1.5 h. Adjusting the pH of the solution to approximately 7 was carried out by using 1 M NaOH solution. As the pH increased, part of chitosan was precipitated. Thereafter, the solution was centrifuged at 6000 rpm for 30 minutes to separate sediments. The upper phase of the centrifuged solution included water-soluble chitosan (WSC), which was soluble in neutral pH, and the lower phase consisted of low molecular weight chitosan (LMC). Then, WSC was vacuum filtered with the aid of appropriate filter paper, while the wet LMWCS was submitted to ultrasonic irradiation with an amplitude of 100 Hz for 20 minutes in order to break the chains further. The final product was dried under vacuum at 40 °C for 48 h and chracterized based upon FTIR, ^{1}H NMR and molecular weight (MW) determination by viscometric measurements using an Ubbelohde Capillary Viscometer (0.5 mm). Average molecular weights were calculated from $[\eta] = k.M^{\alpha}$ equation, where η intrinsic viscosity, k = 1.81 x 10^3 (cm^3/g) and α = 0.93 determined in 0.25M CH_3COONa and 0.25M CH_3COOH solution at 25 °C.

7. Synthesis of polyimidazolium-supported chitosan Schiff base (PICSB)

Generally, 1 g of LMC (*85*% deacetylated, *Alfa Aesar*) was dissolved in 200 mL of a mixed solution of 1% aqueous acetic acid/ethanol under stirring at room temperature for 30 min. A solution of ionic liquids-based salicyldehyde (SIIL, **2**) (equivalent to molar N-content in CS) in ethanol (30 mL) was added to the CS solution over a period of 30 min at 30 °C. Then the reaction mixture was stirred at 40 °C for 24 h. The reaction product was precipitated by adding an excessive amount of ethyl acetate under ultrasonic irradiation at room temperature for 3 h, filtered to remove the solvent and then washed with 30:70, 20:80 and 0:100 ethanol:ethyl acetate mixtures sequentially. Finally, the product was dried at 35 °C under vacuum for 24 h to obtain the desired ionic liquid-functionalized CS Schiff bases (PICSB). It was obtained as a pale yellow powder, Yield (1.63 g, 98.9%).

FTIR (KBr, cm^{-1}): 3427 (vs, br, $\nu_{(OH+NH2)}$), 3146 (m, br, $\nu_{(NH)}$), 1635 (vs, sh, $\nu_{(CO)amide}$+ $\nu_{(CN)Azomethine}$), 1535 (m, sh, amide II), 1383 (m, sh, amide III), 1283 (m, sh, $\nu_{(Ar-O)}$), 1152 (s, sh, $\nu_{(HCC+HCN)bend}$, Im), 1068 (m, sh, $\nu_{(CO-C)str}$), 897 (m, sh, $\nu_{(CO-C)}$, β-glycosidic linkage), 765 (m, sh, Im). ^{1}H NMR (600 MHz, 1% $CD_3COOD/D_2O)_{60\,°C}$ δ (ppm): 9.82 (s, 2H, 2 x OH), 8.55 (dd, *J* = 10.8, 7.7 Hz, 2H, 2 x HCN), 7.80–7.66 (m, 1H, NH of GlcNHAc and Ar-H), 7.65–7.45 (m, 2H, Ar-H), 7.32 (dd, *J* = 14.5, 7.5 Hz, 3H, Ar-H), 7.10 (t, *J* = 7.7 Hz, 1H, Ar-H), 5.51 (d, *J* = 8.4 Hz, 4H, 2 x CH_2 of CH_2Ar), 4.80 (d, *J* = 5.9 Hz, 2H, GlcN residue), 4.09–3.94 (m, 7H, GlcN and GlcNAc residue), 3.80 (s, 6H, 2 x CH_3), 3.26 (br, s, 2H, GlcN residue), 1.96 (s, 6H, 2 x CH_3), 1.13 (t, *J* = 7.2 Hz, 3H, CH_3, NHAc). ^{13}C NMR (151 MHz, 1% $CD_3COOD/D_2O)_{60\,°C}$ δ (ppm): 194.05, 176.9, 167.30, 159.57, 142.58, 134.84, 131.21, 126.51, 124.22, 122.57, 115.67, 110.96, 100.21, 83.89, 77.61, 76.55, 72.36, 66.19, 58.80, 55.01, 53.65, 50.48, 35.96, 31.99, 29.89, 24.32.

8. Synthesis of polyimidazolium-supported amphiphilic chitosan Schiff base (PIACSB)

It was prepared by grafting quaternary ammonium groups onto PICSB surface. Briefly, a mixture containing 1.5 g of PICSB and 9.345 g (61.5 mmol) of glycidyltrimethylammonium chloride (GTMHAC) dispersed in 100 mL of isopropanol was stirred at 70 °C for 30 h. After adding acetone to the reaction mixture, the intended product (PIACSB) was precipitated; the product was then filtered, washed thoroughly with acetone followed by ethanol solution (95%), and dried under vacuum at 50 °C for 24 hours.

9. Antimicrobial activity

7.1. Agar well-diffusion (AWD) method

Three different concentrations of the materials (25, 50 and 150 µg/mL) were tested against the pathogenic bacterial strains. 20 mL of the nutrient agar was poured into Petri dishes and left to solidify, thereafter, wells were constructed using gel puncture and filled with three different concentrations of the tested compounds (30 µL) in three different wells. The zones of inhibition (ZOI, mm), the clear zone around the wells, were measured as a marker of antibacterial activity.

7.2. Colony forming unit (CFU) method

The antimicrobial activities of nanocomposites and Cipro were screened against *E. Coli*, *S. Aureus*, and *P. aeruginosa* by the CFU method. This experiment was performed using a set of three test tubes for each sample. In the foremost tube, a mixture of nutrient broth (NB) (10 mL) and bacterial culture (1.0 mL) was added. The second test tube (treatment tube) contains a mixture of NB (10 mL), bacterial culture (1.0 mL), and a solution of the tested sample (50 µg/mL). The last test tube was filled with a mixture of NB (10 mL) and a sample (50 µg/mL). Then, 3 mL suspension from each sample was diluted to produce an operating (growth control) suspension of 19.3 x 10^6 and 21.5 x 10^6 CFU/mL for *S. aureus* and *E. coli*, respectively. Thereafter, all test tubes were incubated under shaking for 24 h at 30 °C. Afterward, 20 µL of each diluted suspension was added to the agar medium and kept in an incubator at 37 °C for 24 h. Then, the CFU/mL for each plate was examined using the plate count method. The percentage of bacterial colonies reduction (R%) was calculated by the following equation (**Eq. 1**):

$$\mathbf{R\%} = \frac{\mathrm{BC}_{CT} - \mathrm{BC}_{TT}}{\mathrm{BC}_{CT}} \qquad \textbf{(Eq. 1)}$$

where BC_{CT} and BC_{TT} are the number of bacterial colonies in growth control and treatment test tubes, respectively.

7.3. Minimal inhibitory/ bactericidal concentrations (MIC/ MBC)

In brief, adequate dispersions of nanocomposites (NCs) and antibiotics in DMSO were prepared and diluted with MHB to prepare the treatment samples at concentrations ranging from 0.25 µg/mL to 50.0 µg/mL. Thereafter, 190 µL of bacterial suspensions (10^6 CFU/mL) were transferred to microtiter plates with 96 wells and then treated with samples of NCs and antibiotics, separately, at the desired concentration. The plates were then incubated at 37 °C for 24 h; untreated wells (DMSO in MHB is used instead of NBC solution) were used as controls. The turbidity of wells was used as indicative of antibacterial efficacies and was used to calculate MIC and MBC values. Findings of three independent replicates were used and represented as mean ± SD.

10. *In vitro* cytotoxicity activity

By using the MTT assay, the newly synthesized compounds were evaluated for their cytotoxicity in vitro. Briefly, the cell lines were treated with serial doses (1.56, 3.12, 6.25, 12.5, 25, and 50 µg/mL) of solutions of the tested material in a 96-well plate (10^5 cells/well) (Falcon, NJ, USA). DMSO was served as control. After 48 h of incubation at 37 °C in a 5% CO_2 atmosphere, cells in each well were rinsed and stained with an MTT reagent (5 mg/mL in a 0.9% NaCl solution) before being re-incubated for another 4 h. Following incubation, the staining medium was carefully discarded, and 180 µL of acidified isopropanol/well was added to the plate and shaken at room temperature using a MaxQ 2000 plate shaker (Thermo Fisher Scientific Inc., MI, USA) to solubilize the produced formazan crystals. The plate was then spectrophotometrically analyzed by measuring absorbance at 570 nm with a Stat FaxR 4200 plate reader (Awareness Technology, Inc., FL, USA), to estimate the cell viability.

8. Figures

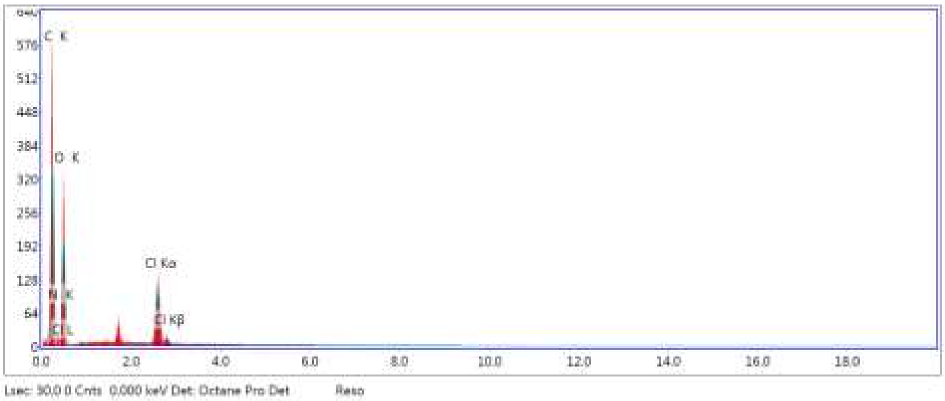

Figure S1: EDS spectrum of PIACSB

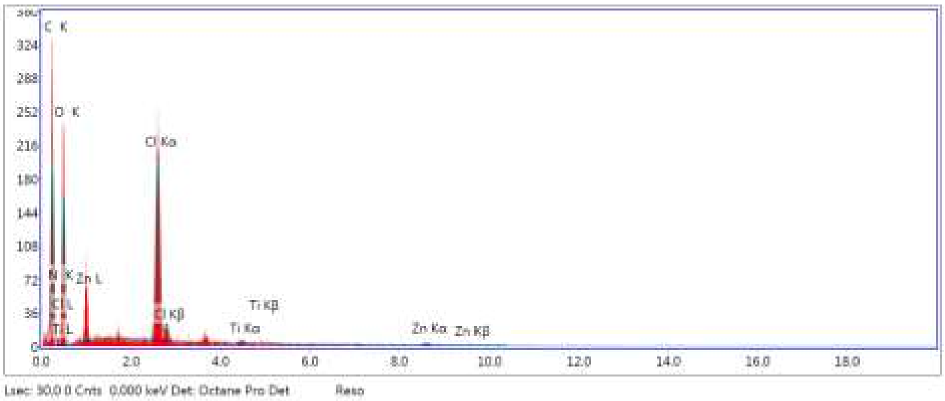

Figure S2: EDS spectrum of ZnO-PIACSB-TiO_2 (**NBC**)

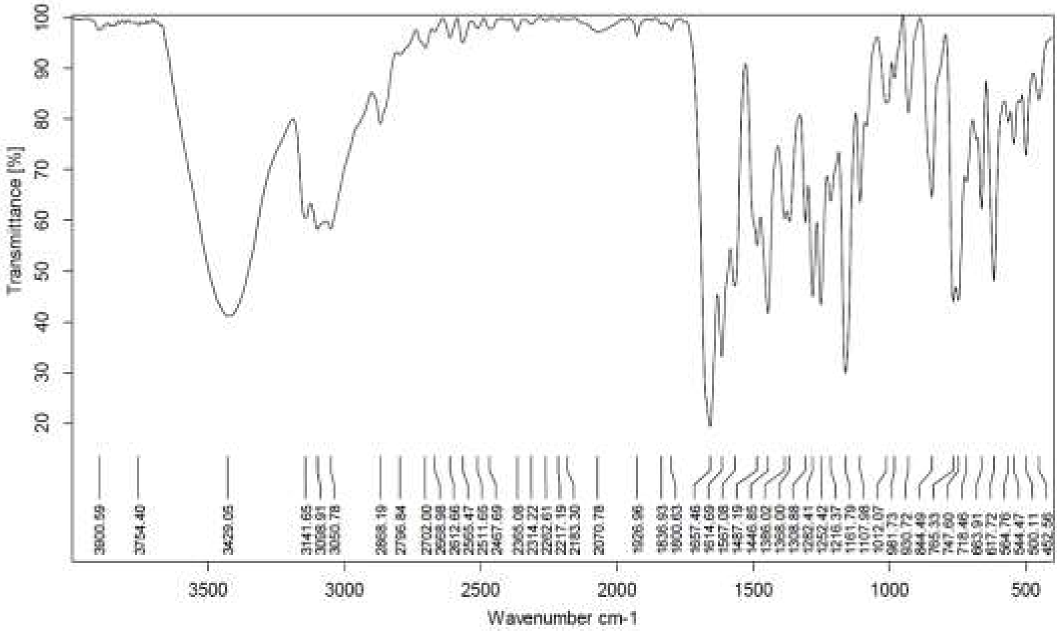

Figure S3: FTIR of SIIL (**2**)

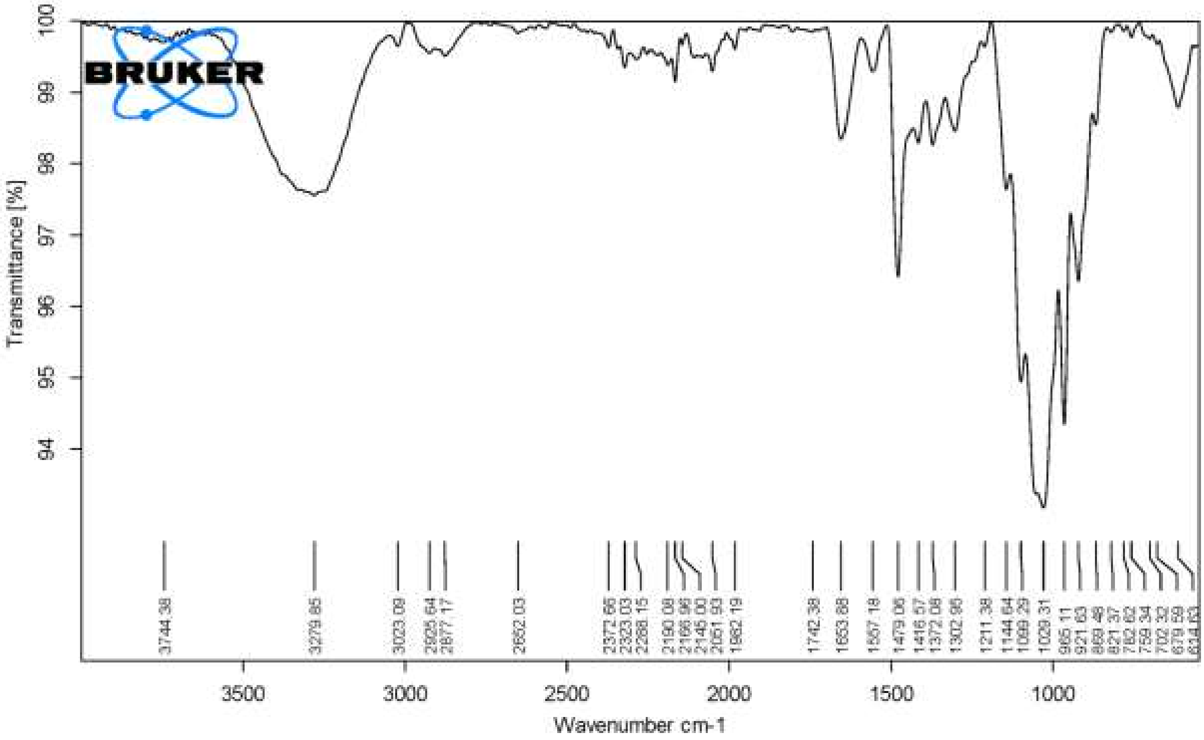

Figure S4: FTIR of LMC

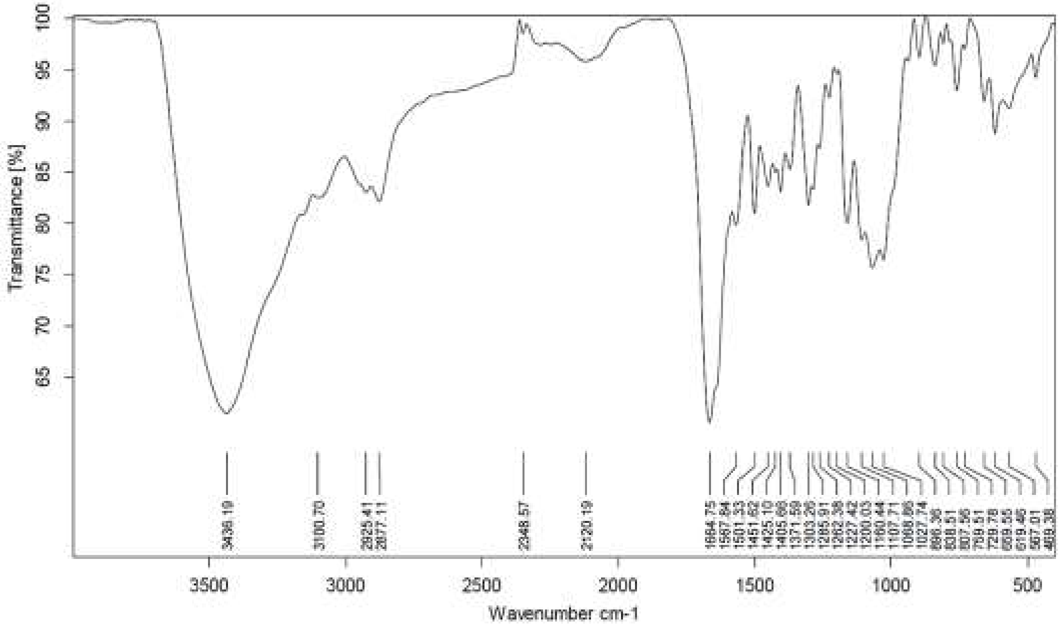

Figure S5: FTIR of PICSB

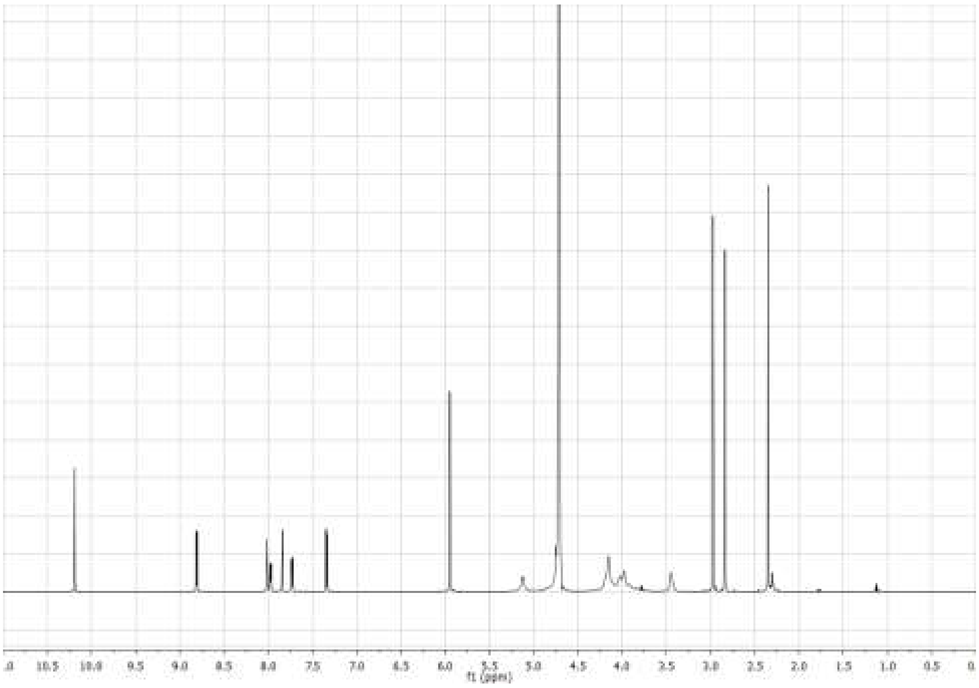

Figure S6: ^{1}HNMR (200 MHz) of PIACSB in D_2O

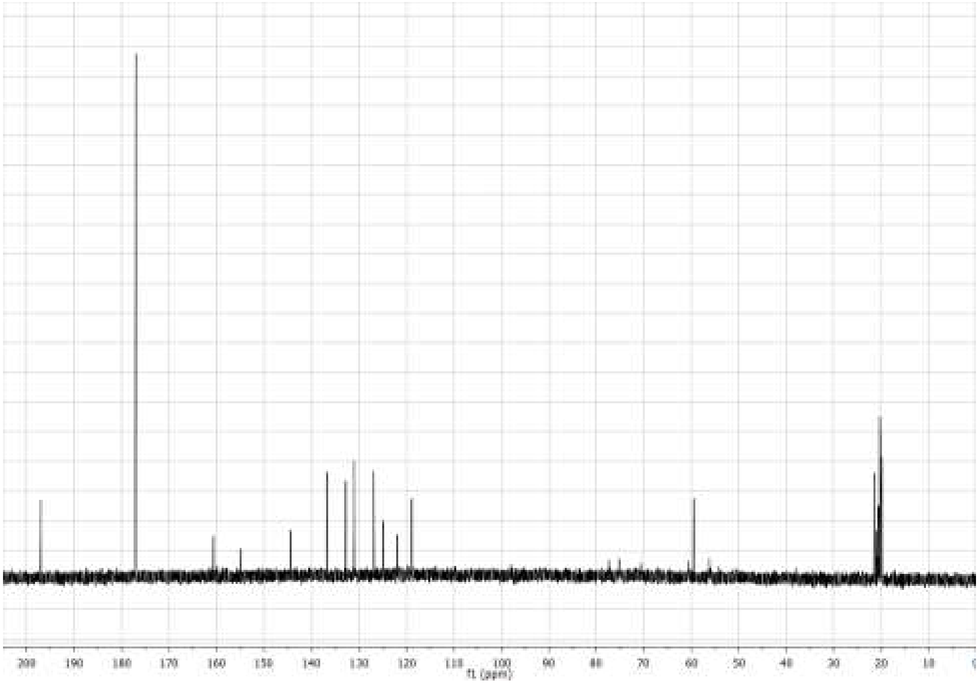

Figure S7: ^{13}CNMR (125 MHz) of PIACSB in D_2O

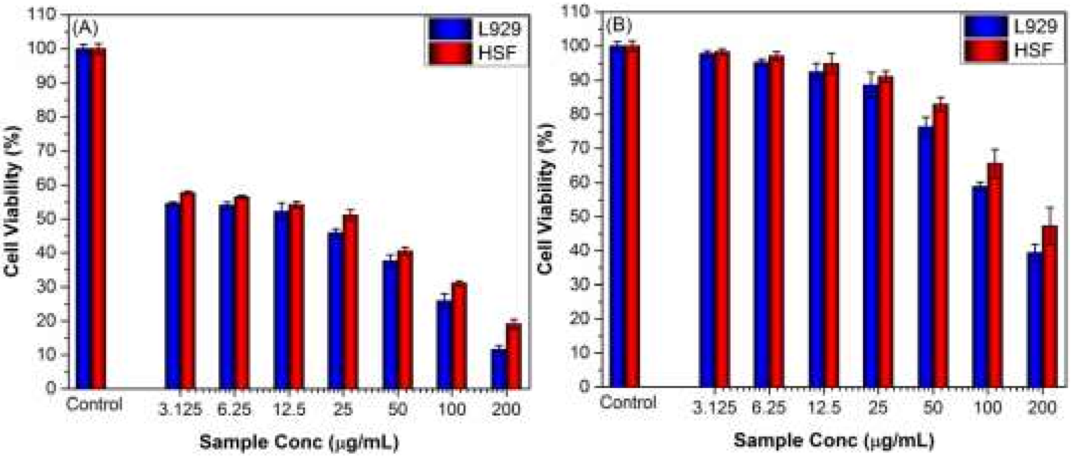

Figure S8: Inhibitory effects of serial doses (3.125-200 μg/mL) of (A) ZnO-TiO_2 and (b) ZnO-PIACSB-TiO_2 on the proliferative activities of L929 and HSF cells

Table S1: Molecular structure indices (M_{av}, EA, DD, and QD) of new stabilizers (SIIL and PIACSB) and TiO_2-ZnO nanocomposites

Sample	M_{av} (kDa)	EA%				DD%			QD%	ζ-potential (mV)	MPD (nm)
		C	H	N	Ru	EA	Tit.	^{1}H-NMR			
SIIL	NA	58.12	7.07	8.35	NA	NA	NA	NA	NA	+49.15	NA
LMC	24.5	45.03	6.86	8.38	NA	88.9	84.7	87.7	NA	+18.76	NA
PIACSB	39.37	57.82	9.98	6.29	NA	NA	NA	NA	81.1	+43.22	NA
TiO_2-ZnO	NA	42.76	6.15	7.12	16.53	NA	NA	NA	NA	+39.95	27.97
NBC	NA	46.32	5.36	7.98	19.92	NA	NA	NA	NA	+48.07	51.01

Table S2: Antimicrobial NBC vs. previous TiO_2-ZnO-based nanocomposites

Sample	*E. coli*		*S. aureus*		*P. aeruginosa*		*A. flavus*		Ref.
	MIC	MBC	MIC	MBC	MIC	MBC	MIC	MFC	
**TiO_2/ZnO/4Az**	1 ± 0.01[a]	2 ± 0.01[a]	2 ± 0.01[a]	3 ± 0.01[a]	–	–	–	–	[58]
**ZC/T0.5[c]**	24.56[b]	–	56.34[b]	–	–	–	–	–	[59]
**ZC/T0.7[d]**	22.78[b]	–	15.32[b]	–	–	–	–	–	[59]
**ZTCC HNM[e]**	250[b]	500[b]	250[b]	500[b]	–	–	–	–	[60]
**TiO_2/AgNCs**	31.2[b]	31.2[b]	62.5[b]	62.5[b]	31.2[b]	31.2[b]	–	–	[61]
**ZnO/TiO_2/AgNCs**	15.8[b]	15.8[b]	31.2[b]	31.2[b]	9.6[b]	9.6[b]	–	–	[61]
**NBC**	0.20[b]	0.40[b]	0.34[b]	0.68[b]	0.15[b]	0.30[b]	–	–	This work

[a] mg/mL

[b] μg/mL

[c] ZnO/natural clay/TiO_2

[d] ZnO/natural clay/TiO_2

[e] Hybrid nanomaterial composed of chitosan, curcumin, ZnO and TiO_2
